# Competitive Semantic Memory Retrieval: Temporal Dynamics Revealed by Event-Related Potentials

**DOI:** 10.1371/journal.pone.0150091

**Published:** 2016-02-22

**Authors:** Robin Hellerstedt, Mikael Johansson

**Affiliations:** Department of Psychology, Lund University, Lund, Sweden; Goethe-Universitat Frankfurt am Main, GERMANY

## Abstract

Memories compete for retrieval when they are related to a common retrieval cue. Previous research has shown that retrieval of a target memory may lead to subsequent retrieval-induced forgetting (RIF) of currently irrelevant competing memories. In the present study, we investigated the time course of competitive semantic retrieval and examined the neurocognitive mechanisms underlying RIF. We contrasted two theoretical accounts of RIF by examining a critical aspect of this memory phenomenon, namely the extent to which it depends on successful retrieval of the target memory. Participants first studied category-exemplar word-pairs (e.g. Fruit—Apple). Next, we recorded electrophysiological measures of brain activity while the participants performed a competitive semantic cued-recall task. In this task, the participants were provided with the studied categories but they were instructed to retrieve other unstudied exemplars (e.g. Fruit—Ma__?). We investigated the event-related potential (ERP) correlates of retrieval success by comparing ERPs from successful and failed retrieval trials. To isolate the ERP correlates of continuous retrieval attempts from the ERP correlates of retrieval success, we included an impossible retrieval condition, with incompletable word-stem cues (Drinks—Wy__) and compared it with a non-retrieval presentation baseline condition (Occupation—Dentist). The participants’ memory for all the studied exemplars was tested in the final phase of the experiment. Taken together, the behavioural results suggest that RIF is independent of target retrieval. Beyond investigating the mechanisms underlying RIF, the present study also elucidates the temporal dynamics of semantic cued-recall by isolating the ERP correlates of retrieval attempt and retrieval success. The ERP results revealed that retrieval attempt is reflected in a late posterior negativity, possibly indicating construction of candidates for completing the word-stem cue and retrieval monitoring whereas retrieval success was reflected in an anterior positive slow wave.

## Introduction

Memories that are associated with a common retrieval cue are reactivated and compete for retrieval when the shared cue is presented. An everyday-example of such competitive cued recall is when someone asks you about your friend’s address. In this situation, the memory of her current address, the target-memory, and memories of previous addresses, competitors, will compete for retrieval. Memory research has suggested that the ability to retrieve the currently relevant target-memory comes at a cost, namely forgetting of the competing memories [1]. The phenomenon that competitive retrieval causes forgetting of related memories is referred to as retrieval-induced forgetting (RIF; for reviews see [2–4]. There is an on-going debate regarding the cognitive mechanisms underlying RIF. The associative blocking account holds that retrieval of the target memory strengthens the association between the target memory and the retrieval cue. The next time the retrieval cue is presented the target memory is more likely to be reactivated than the competitors given that it has a stronger association to the retrieval cue. In this way, the associative blocking account proposes that successful target retrieval causes blocking of competing memories in ensuing retrieval situations involving the same retrieval cue [4]. Another theory, the inhibitory control account [1] has challenged the associative blocking account. According to this theory, inhibitory control is recruited to inhibit currently irrelevant competing memories in order to facilitate retrieval of the relevant target memory. In a previous study, we investigated the ERP correlates of competitor activation and the role of this process in RIF [5]. We here continue this line of research by examining the role of target retrieval for RIF with behavioural and electrophysiological methods. The reason for investigating the relation between target retrieval and RIF is that the two earlier described theoretical accounts of RIF have opposite predictions regarding the relationship between target retrieval and RIF; more specifically, concerning the role of target retrieval success.

The experimental paradigm used to investigate RIF (i.e. the retrieval-practice paradigm) typically involves three phases [1]. In the initial encoding phase, the participants study category-exemplar word-pairs (e.g. Fruit—Mango, Fruit—Kiwi, Occupation—Dentist, Occupation—Policeman). A retrieval-practice phase follows the encoding phase in which participants practice retrieval of half of the exemplars from half of the studied categories (e.g. Fruit—Ma___?). In the third and final phase of the paradigm, the participants are tested on all the exemplars that were included in the study phase. RIF refers to the finding that memory performance is reduced for non-practiced items from practiced categories (e.g. Fruit—Kiwi) compared with non-practiced items from non-practiced categories (e.g. Occupation—Dentist).

The associative blocking account (e.g. [4]) posits that RIF is *dependent* upon successful target retrieval in the intermediate retrieval-practice phase. This theory holds that target retrieval strengthens the association between the cue and the target (e.g. Fruit and Mango). When the category-cue (Fruit) is later represented in the final recall test, it will activate the strengthened exemplars (Mango) and block retrieval of the non-strengthened (non-practiced) memories associated with the same cue (e.g. Kiwi). In contrast, the inhibitory control account assumes that RIF is *independent* of target retrieval success in the retrieval-practice phase. This theory postulates that presentation of the category-plus-word-stem cue (Fruit–Ma__?) reactivates studied exemplars belonging to the category (e.g. Mango and Kiwi) and that cognitive control mechanisms are recruited to inhibit the currently irrelevant competitors (e.g. Kiwi) to facilitate retrieval of the target memory (Mango). The inhibition of the competitors causes lowered accessibility and forgetting of these exemplars in the ensuing final recall test.

Previous research has lent support to the notion that RIF is independent of target retrieval. In fact, in two studies, RIF has even been observed in a task where retrieval-practice is impossible and there can be no target memory strengthening [6,7]. Participants were provided with category-plus-word-stem cues that did not match any category exemplar, making the retrieval task impossible (e.g. Drinks—Wy___?). The present study combined an impossible retrieval-practice task with electrophysiological measures of brain activity to further specify the relationship between target retrieval and RIF and to investigate the neurocognitive mechanisms involved in competitive retrieval and associated ensuing RIF. An advantage of the ERP approach is that it makes it possible to investigate the mechanisms underlying RIF as they occur in the retrieval-practice phase rather than studying the consequences of these mechanisms indirectly in a subsequent memory-test as is done with behavioural measures only.

A semantic version of the retrieval-practice task was used in the present study. In this task, participants were given the same category-plus-word-stem cue as in the standard episodic retrieval-practice task, but they were asked to retrieve an exemplar from semantic long-term memory instead of an exemplar that had been presented in the encoding phase (none of the studied exemplars matched the word stem cues). This semantic competitive-retrieval task has been shown to induce RIF in previous studies [5–8], and was used here for two reasons: First, it effectively obscured the fact that the word-stem cues in the impossible retrieval condition were incompletable, since it made the number of possible candidates for completing the cue unknown rather than restricted to the previously studied exemplars. Second, it allowed us to investigate and isolate the ERP correlates of retrieval attempt and retrieval success in semantic cued recall, which have only received limited attention in the ERP memory literature thus far.

The three conditions in the present experiment differed only in the competitive semantic-retrieval phase, where participants were provided with either a completable retrieval cue (possible retrieval condition; Fruit—Ma__? Mango), an incompletable retrieval cue (impossible retrieval condition; Drinks—Wy__?), or an intact category-exemplar word-pair (presentation baseline condition; Occupation—Dentist). The associated tasks were to retrieve an exemplar from semantic memory that completed the word-stem cue when a word-stem was given (possible and impossible retrieval conditions) and to read the exemplar when an intact exemplar was presented (presentation baseline condition), i.e. only the former two tasks involved memory retrieval.

We examined the relationship between target retrieval and RIF with behavioural measures in two ways: a) if unsuccessful retrieval attempt causes forgetting in the impossible retrieval condition, and b) if target retrieval in the possible retrieval-practice condition predicted RIF in the ensuing test.

Importantly, the inhibitory control account and the associative blocking account have opposite predictions regarding the outcome of these two tests. According to the blocking account, RIF is dependent on target retrieval and this theory hence predicts that there should be no RIF in the impossible retrieval condition. This theory also predicts that there should be a positive correlation between target retrieval success in the competitive semantic retrieval phase and RIF in the episodic final test. On the contrary, the inhibitory control account holds that RIF is independent of target retrieval and predicts that a mere retrieval attempt is sufficient to induce forgetting without the prerequisite of retrieval success. Only one research group so far has reported RIF in an impossible retrieval-practice task [6], [7]. We here aimed to replicate this finding with a slightly different setup (e.g. a different baseline as described below).

In addition to these behavioural tests of the role of target retrieval in RIF (i.e. strength dependence), we examined the relationship between RIF and the ERP correlates of retrieval attempt and retrieval success in competitive semantic retrieval. The logic of the ERP analyses was that ERP correlates of retrieval attempt should be evident in contrasts between failed retrieval trials (the impossible retrieval condition and retrieval failure trials in the possible retrieval condition) and the presentation baseline condition, whereas ERP correlates of retrieval success should be evident in comparisons between successful retrieval trials and failed retrieval trials within the possible retrieval condition. The two accounts of RIF have opposite predictions regarding the relationship between RIF and these ERP correlates. The associative blocking account assumes that RIF is dependent upon target retrieval and hence predicts a positive correlation between RIF and the ERP correlates of retrieval success, but no such correlation between RIF and ERP correlates of retrieval attempt. The inhibitory control account, on the other hand, assumes that RIF is independent of target retrieval and predicts no correlation between RIF and ERP correlates of retrieval success. Inhibitory control is theorized to be recruited to handle interference during competitive retrieval attempts even if retrieval is unsuccessful. The inhibitory control account accordingly suggests that potential ERP correlates of inhibitory control should be evident in the retrieval attempt ERP contrast and that an index of inhibitory control will be positively related to RIF. Previous research on inhibitory control during memory retrieval has suggested that inhibition is reflected in enhanced N2 amplitude (e.g. [9–11]). This N2 effect is typically maximal over frontocentral electrode sites and occurs approximately 300–500 ms after the retrieval cue has been presented. Thus, in the present study, we predict that an N2 effect will be evident in the retrieval attempt contrast and that this effect will predict increased levels of RIF in the final test.

The present study investigated the temporal dynamics of competitive retrieval in a *semantic* cued recall task. Previous ERP studies of cued recall have predominantly focused on *episodic* rather than on semantic memory (e.g. [12], [13]). In fact, semantic cued-recall has often been used as a baseline in these studies (e.g. [12], [14–17]). The typical finding in these studies have been that successful episodic compared with semantic retrieval is associated with more positive going ERPs starting approximately 400 milliseconds (ms) post cue presentation and lasting until the end of the recording epoch (e.g. [12], [14], [16–19]). This positive slow wave (PSW) effect is typically maximal over anterior electrode sites. Retrieval from semantic memory has been related to a similar PSW effect, although the topography and timing have varied between tasks and studies [5], [20], [21]. Rass and colleagues (2010) observed that competitive word-fragment completion, i.e. semantic retrieval in the face of a primed similar but non-matching memory, was related to an anterior bilateral PSW onsetting 500 ms after stimulus presentation and lasting until the end of the recording epoch. Cansino and colleagues (1999) reported that solving the scrabble task was related to a right lateralized PSW between 1000 and 1500 ms post stimulus presentation. We recently related retrieval success to a widespread PSW effect with a bilateral posterior maximum, onsetting approximately 600 ms post cue presentation and lasting until the end of the recording epoch, in the same semantic category-plus-word-stem cued-recall task (e.g. “Fruit—Ma___?” for Mango) used in the present study [5]. In summary, the retrieval success related PSW effect typically onsets 400 to 600 ms post cue presentation. The topography of the PSW has varied between previous studies.

Besides investigating the ERP correlates of *semantic* retrieval success we will also examine the ERP correlates of retrieval success in *episodic* cued recall in the final episodic test. By comparing the topographies of the two retrieval success ERP effects we will test if separate neural generators underlie retrieval from semantic and episodic memory.

Given that no prior studies have investigated the ERP correlates of competitive *semantic retrieval attempts*, we base our predictions on previous studies of *episodic* cued recall. One candidate ERP correlate of semantic retrieval attempt is the late posterior negativity (LPN). The LPN ERP effect has been related to a) the continuous reconstruction and evaluation of attribute conjunctions (e.g. item-source or item-context associations) when such conjunctions are not readily recovered by the test probe, and to b) error monitoring mechanisms that are engaged during memory retrieval (for review of the LPN see [22]). Both of these proposed functional characteristics of the LPN are likely to be relevant also in semantic cued recall. In order to complete the category-plus-word-stem cue, the participants need to construct candidate exemplars to evaluate. This could be achieved by retrieval of lexical representations that together generate a valid exemplar (i.e. retrieval of attribute conjunctions). Moreover, retrieval monitoring should be recruited continuously during the semantic competitive-retrieval task employed in the present experiment as the participant retrieve exemplars and match them to the category-plus-word-stem cue. In the case of the impossible retrieval condition, we expect such monitoring, including error-detection, and continued search to continue throughout the epoch.

To summarize, the present study contrasted two prominent theoretical accounts of RIF by evaluating the role of target retrieval. Beyond investigating the mechanisms underlying RIF, the present study aimed at furthering our understanding of the temporal dynamics of semantic cued-recall by isolating ERP correlates of retrieval attempt and retrieval success and by comparing ERP correlates of semantic and episodic memory retrieval success.

## Methods

### Participants

18 participants (9 females) gave written informed consent before participating in exchange for a cinema ticket. All participants had normal or corrected to normal vision, were native Swedish speakers and were right handed. Mean age was 23.6 (*SD* = 4.678, range 21–37). After the experiment, the participants were debriefed about the purpose of the study and informed that impossible retrieval cues were included in the competitive semantic retrieval phase. The study was approved by the regional ethics committee at Lund University.

### Materials

We selected thirty distinct semantic categories from the Swedish category norms [23]. For each category, we selected 12 exemplars. Six exemplars were presented in the encoding phase and the remaining six were used as targets in the competitive semantic retrieval phase of the experiment. The exemplars were between four and ten letters long. All exemplars had a unique initial letter within their category. We validated the incompletable word-stems in the impossible retrieval condition via searches in a Swedish dictionary [24].

### Design

The experiment consisted of five blocks. Each block was divided into four phases: encoding, competitive semantic retrieval, distracter task (digit stroop) and test (cf. [5]). Six categories were included in each experimental block. These categories were assigned to the three conditions (two categories per condition in each block) and this assignment of categories to condition was counterbalanced across participants.

### Procedure

**Fig 1:**
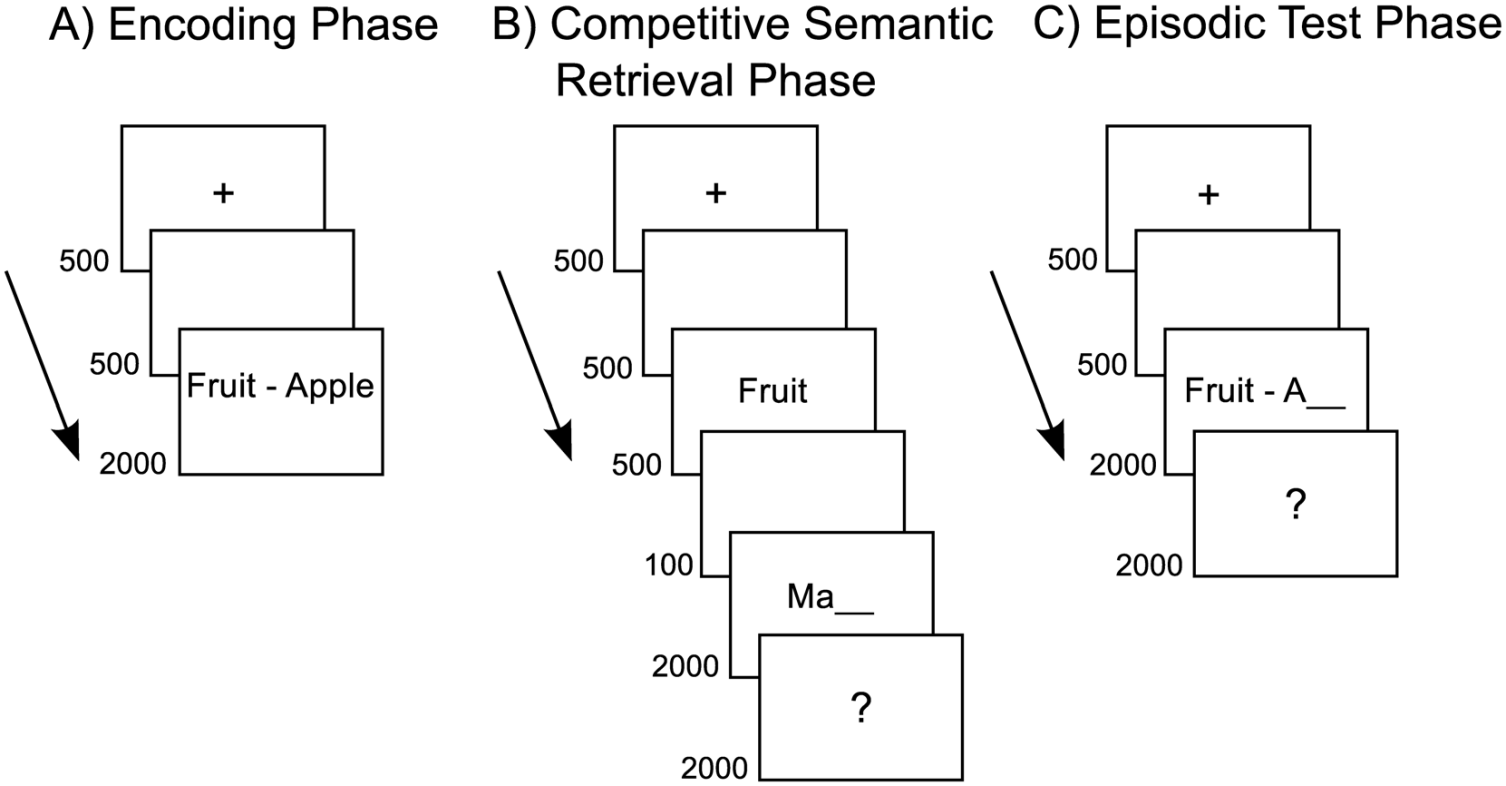
Trial procedures in the three phases of the experiment. Presentation durations are displayed in milliseconds.

The trial structure in each phase of the experiment is depicted in Fig 1.

#### Encoding phase

Thirty-six category-exemplar word-pairs from six categories were presented in black font colour on a white background (e.g. Fruit—Orange) in each block. The participants were instructed to memorize the presented word pairs. Each trial began with a 500 ms presentation of a fixation cross followed by a 500 ms presentation of a white screen before the category exemplar word pair was shown for 2000 ms.

#### Competitive semantic retrieval

The encoding phase was followed by a competitive semantic retrieval phase. This phase was the only part of the experiment in which the conditions differed from each other. In the possible retrieval condition, a category and a word-stem cue were presented sequentially and the task was to retrieve an exemplar that completed the category-plus-word-stem cue. All the categories had been presented in the encoding phase. Importantly, to make it a semantic rather than an episodic retrieval-task, none of the studied exemplars in the encoding phase matched the category-plus-word-stem cue in the competitive semantic retrieval phase. The participants were informed that the encoded exemplars would not match the cue and were instructed to retrieve another exemplar from semantic long-term memory that completed the presented category-plus-word-stem cue (e.g. Fruit—Ma___, Mango). In the impossible retrieval condition, participants were presented with a word-stem that did not match any exemplar in the category (e.g. Fruit—Tu___,?), making the semantic retrieval-task impossible. All participants reported in the debriefing that they were unaware of the fact that impossible word stems were included in the experiment. In the presentation baseline condition, the participants were provided with an intact exemplar rather than a word stem and the task was to read the presented exemplar and to say it aloud at the end of the trial (when a question mark indicated that they should respond). Each trial started with a 500 ms presentation of a fixation-cross presented at the centre of the screen. The fixation cross was followed by a 500 ms presentation of a blank screen before a category cue was presented on the screen for 500 ms (e.g. Fruit). Next, a blank screen was shown for 100 ms before the word-stem cue (or alternatively a complete exemplar in the presentation baseline condition) was presented for 2000 ms. Each trial ended with a 2000 ms presentation of a question mark. We instructed the participants to withhold their response until the question mark was presented to avoid muscle artefacts in the ERP recording epoch. We also told the participants to say “pass” when they did not manage to retrieve an exemplar that matched the cue, to ensure that the participants prepared an oral response in all conditions. After the competitive semantic retrieval phase, the participants engaged in a distracter task (digit stroop) for approximately five minutes.

#### Final episodic test

A cued-recall test of all studied exemplars followed the distracter task. As in the previous phases of the experiment, each trial began with a 500 ms presentation of a fixation cross, followed by a 500 ms presentation of a blank screen. Next, a category-plus-initial-letter cue was presented (e.g. Fruit—O___) for 2000 ms followed by a 2000 ms presentation of a question mark. The participants were instructed to covertly retrieve an exemplar from the encoding phase of the experiment that completed the cue and to respond orally when the question mark was shown on the screen.

### Electroencephalogram Recording and Preprocessing

The electroencephalogram (EEG) was recorded from 35 silver/silver chloride electrodes mounted in an elastic cap. The vertical and horizontal electrooculogram were recorded from four additional electrodes placed over and below the left eye and at the left and right outer canthi. All channels were digitized with a 24-bit resolution at a sample rate of 512 Hz with a Neuroscan Synamps 2/RT amplifier (Compumedics, El Paso, TX, USA). The preprocessing and analysis of the EEG data was performed in Matlab using the EEGlab toolbox [25], the ERPlab toolbox [26], and self-written code. The EEG was referenced to the left mastoid during the recording and re-referenced off-line to the average of the left and the right mastoids. The scalp-electrode impedances were kept below 5 kΩ throughout the recording. A 0.1–30 Hz (12 decibel/octave) band-pass filter was applied to the EEG off-line to increase the signal-to-noise ratio. The continuous EEG was segmented into epochs beginning 200 ms prior to the presentation of the category cue and ending 2500 ms after the presentation of the same stimulus. The prestimulus interval was used for baseline correction of the ERPs. Electro-oculogram artefacts were corrected using the independent component analysis (ICA) procedure in the EEGlab toolbox [25]. Epochs containing recording related artefacts were rejected prior to averaging. The average number of accepted trials from the competitive semantic retrieval phase was 53.4 (*SD* = 6.5, range = 37–60) in the possible retrieval condition, 52.3 (*SD* = 8.7, range = 30–60) in the impossible retrieval condition, and 53.0 (*SD* = 6.9, range = 35–59) in the presentation baseline condition. Within the possible retrieval condition, an average number of 27.3 (*SD* = 5.8, range = 16–35) and 26.2 (*SD* = 4.7, range = 19–39) trials were accepted for retrieval success and retrieval failure, respectively. Finally, in the final episodic recall test the average number of accepted trials was 54.4 (*SD* = 12.8, range = 26–87) for retrieval success trials and 92 (*SD* = 20.8, range = 61–126) for retrieval failure trials.

### Event-Related Potential Data Analysis

To quantify the ERP waveforms, we calculated mean amplitudes in six consecutive time windows (0–300, 300–600, 600–1000, 1000–1500, 1500–2000 and 2000 2500 ms). These time windows were used in the analysis of data from both the competitive semantic retrieval phase and the final episodic test phase. In the competitive semantic retrieval phase, the time windows can be divided into two category cue time windows (0–300 and 300–600 ms) and four word-stem cue time-windows (600–1000, 1000–1500, 1500–2000 and 2000–2500 ms), whereas the category-plus-initial letter cue was presented during all six time windows in the final episodic test phase. The time windows were labelled in relation to the cue that the ERPs were time-locked to (the category-cue in the competitive semantic retrieval phase and the category-plus-initial-letter cue in the final episodic test). We conducted a Condition (competitive semantic retrieval phase: impossible retrieval/presentation baseline/retrieval success/retrieval failure; final episodic test: retrieval success/retrieval failure) x Region (frontal: F3, FZ, F4/central: C3, CZ, C4/parietal: P3, PZ, P4/occipital: O1, OZ, O2) x Hemisphere (left/ midline/right) omnibus repeated measures analysis of variance (ANOVA) in each time window in both the competitive semantic retrieval phase and the final episodic test phase. Significant main effects or interactions involving Condition were followed up with planned comparisons. First, we investigated the ERP correlates of retrieval attempt by contrasting the impossible retrieval and retrieval failure ERPs with presentation baseline ERPs from the competitive semantic retrieval phase. Next, we compared successful retrieval and retrieval failure ERPs to examine the ERP correlates of retrieval success both for semantic retrieval and episodic retrieval. Greenhouse-Geisser adjustment was used when data violated the assumption of sphericity, as indicated by Mauchly’s test of sphericity.

#### Analysis of the relationship between event-related potential effects and retrieval-induced forgetting

Finally, we examined the relationship between the ERP effects observed during competitive semantic retrieval and RIF in the final episodic test to evaluate predictions from the inhibitory control account and the associative blocking account of RIF. We calculated ERP amplitude differences between the relevant ERP conditions in the time windows and at the electrode sites where the ERP effect was reliable. Next, we calculated a forgetting index separately for the possible and the impossible retrieval conditions. These indices were computed by subtracting the average recall performance in each of the retrieval conditions from the average performance in the presentation baseline condition. To control for differences in baseline performance, we divided the indices by the average performance in the presentation baseline condition (cf. [27]). We analysed the relationship between the ERP amplitude differences and the forgetting indices with Spearman correlation.

## Results

The data are available as S1 Dataset.

### Behavioural Results

#### Competitive semantic retrieval

Mean performance in the competitive semantic retrieval phase was 50.1% (*SD* = 7.1) in the possible retrieval condition and 3.5% (*SD* = 2.1) in the impossible retrieval condition. The participants were thus able to retrieve an exemplar that completed the cue in 3.5% of the supposedly impossible retrieval condition. Some of these responses were accurate and creative word-stem completions that we had not anticipated. Other responses in the impossible conditions were incorrect either in terms of semantic category (an exemplar from another semantic category that completed the word-stem) or spelling (an exemplar belonging to the correct category that did not match the word-stem). We excluded these ‘successful’ impossible retrieval trials from the ERP analyses (both correct and incorrect ones). As expected, a one-way repeated measures ANOVA with the factor Condition (possible retrieval/impossible retrieval) confirmed that the participants were significantly more successful in retrieving exemplars that completed the word-stems in the possible compared to the impossible condition (*F*(1,17) = 756.959, *p* < .001, η^2^_p_ = .978).

#### Final episodic test

Average performance in the final episodic test was 34.3% (*SD =* 9.2) in the possible retrieval condition, 36.9% (*SD* = 8.7) in the impossible retrieval condition, and 40.4% (*SD* = 9.7) in the presentation baseline condition. A one-way repeated measures ANOVA with the factor Condition (Impossible retrieval/Possible retrieval/Presentation baseline) revealed a significant main effect (*F*(2,34) = 6.835, *p =* .003, η^2^_p_ = .287). Consistent with the inhibitory control account, pairwise comparisons revealed reliable reduction in memory performance compared with the presentation baseline condition in both the possible (*F*(1,17) *=* 11.688, *p* = .003, η^2^_p_ = .407) and the impossible retrieval conditions (*F*(1,17) = 5.112, *p* = .037, η^2^_p_ = .231), and there was no significant difference in memory performance between the two retrieval conditions (*F*(1,17) = 2.628, *p* = .123, η^2^_p_ = .134). Moreover, in line with the target retrieval independence of RIF, there was no correlation between retrieval performance in the competitive semantic retrieval task and RIF in the possible retrieval condition (*r*_s_ = .296, *p* = .233).

### ERP Results

**Fig 2:**
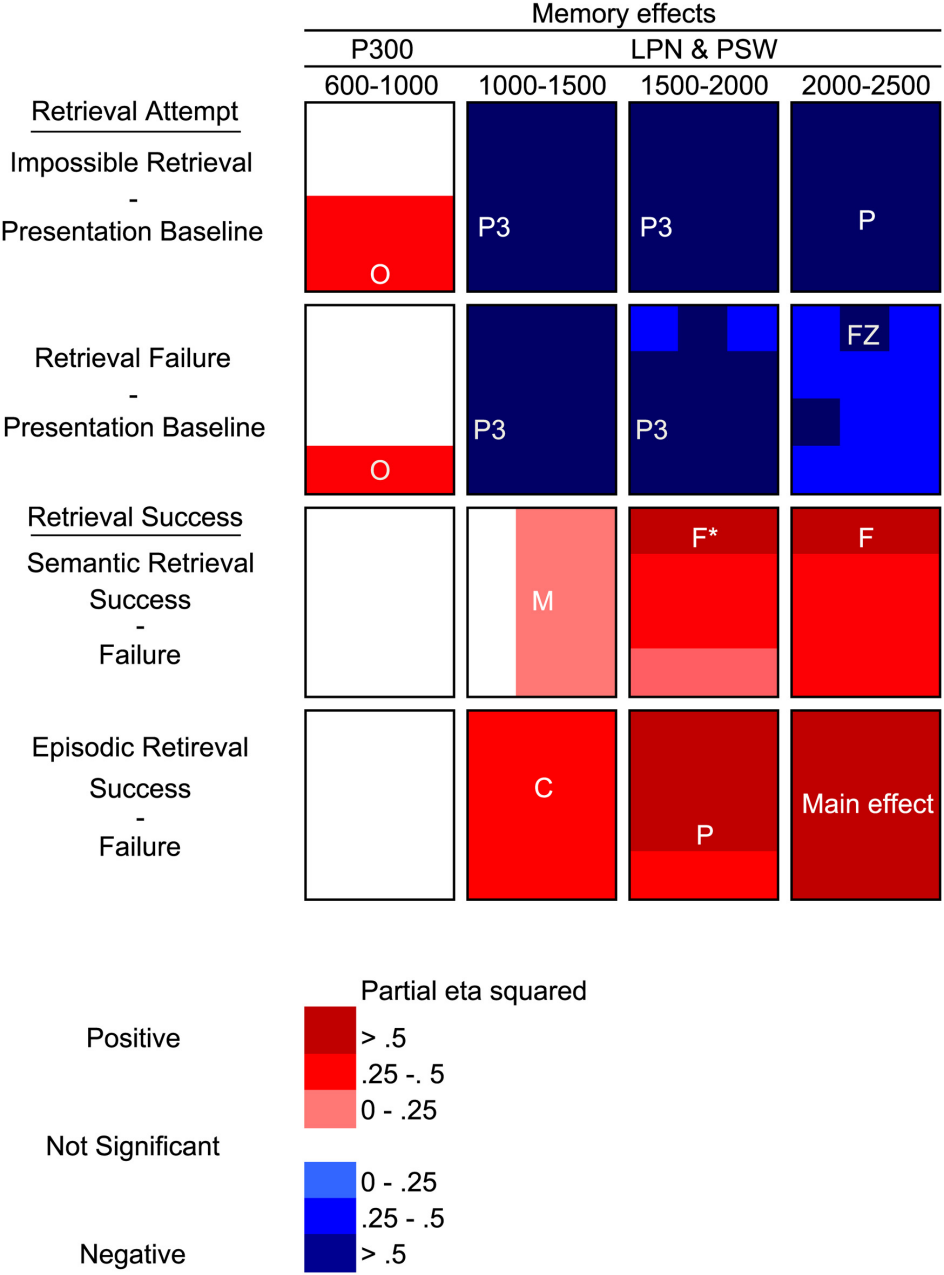
Results from the follow-up analyses of significant interactions from the word-stem cue time windows. Only significant interactions involving the factor Condition were followed up. The magnitude and the direction of the effects in each region are illustrated using a colour scale. The electrode position where the effects had maximal effect size (η^2^_p_) is written in white type font for three way interactions. Similarly, the region with maximal effect size is written in white type fonts for two way interactions. Abbreviations: F = frontal, C = central, P = parietal, O = occipital, M = midline. * In addition to the depicted Condition x Anterior/posterior interaction there was a Condition x Hemisphere interaction in this time window which was maximal in the right hemisphere.

There were no effects involving the factor Condition in the two category-cue time-windows in the omnibus ANOVAs (all *p*s ≥ .125). All effects described below are hence in the four word-stem cue time-windows (600–1000, 1000–1500, 1500–2000 and 2000–2500 ms). The results from the omnibus ANOVAs and the planned comparisons ANOVAs are displayed in Table 1. Fig 2 shows planned follow-up tests of significant effects involving the factor Condition.

**Table 1:** Results from the omnibus and the planned comparison ANOVAs in the word-stem cue time windows.

Effects	Time Windows			
Omnibus	600–1000	1000–1500	1500–2000	2000–2500
Cond	.285 *NS*	< .001	< .001	< .001
Cond x Anterior/posterior	.022	.002	< .001	.026
Cond x Hemisphere	.761 *NS*	< .001	.010	.198 *NS*
Cond x Anterior/posterior x Hemisphere	.089 *NS*	< .001	.023	.057 *NS*
**Impossible vs Presentation Baseline**				
Cond	.049	< .001	< .001	< .001
Cond x Anterior/posterior	.049	< .001	< .001	.029
Cond x Hemisphere	.749 *NS*	.002	.068 *NS*	.585 *NS*
Cond x Anterior/posterior x Hemisphere	.086 *NS*	< .001	.005	.151 *NS*
**Failure vs Presentation Baseline**				
Cond	.354 *NS*	< .001	< .001	< .001
Cond x Anterior/posterior	.047	.007	.005	.075 *NS*
Cond x Hemisphere	.981 *NS*	.017	.063 *NS*	.153 *NS*
Cond x Anterior/posterior x Hemisphere	.091 NS	< .001	.008	.009
**Semantic Retrieval: Success vs Failure**				
Cond	.849 *NS*	.059 *NS*	.002	.001
Cond x Anterior/posterior	.331 *NS*	.276 *NS*	.015	.026
Cond x Hemisphere	.420 *NS*	.013	.006	.077 *NS*
Cond x Anterior/posterior x Hemisphere	.362 *NS*	.273 *NS*	.214 *NS*	.136 *NS*
**Episodic Retrieval: Success vs Failure**				
Cond	.711 *NS*	.002	< .001	< .001
Cond x Anterior/posterior	.394 *NS*	.010	.041	.121 *NS*
Cond x Hemisphere	.196 *NS*	.056 *NS*	.164 *NS*	.573 *NS*
Cond x Anterior/posterior x Hemisphere	.322 *NS*	.095 *NS*	.687 *NS*	.840 *NS*

The α-level was set to .05.

#### Semantic retrieval attempt

**Fig 3:**
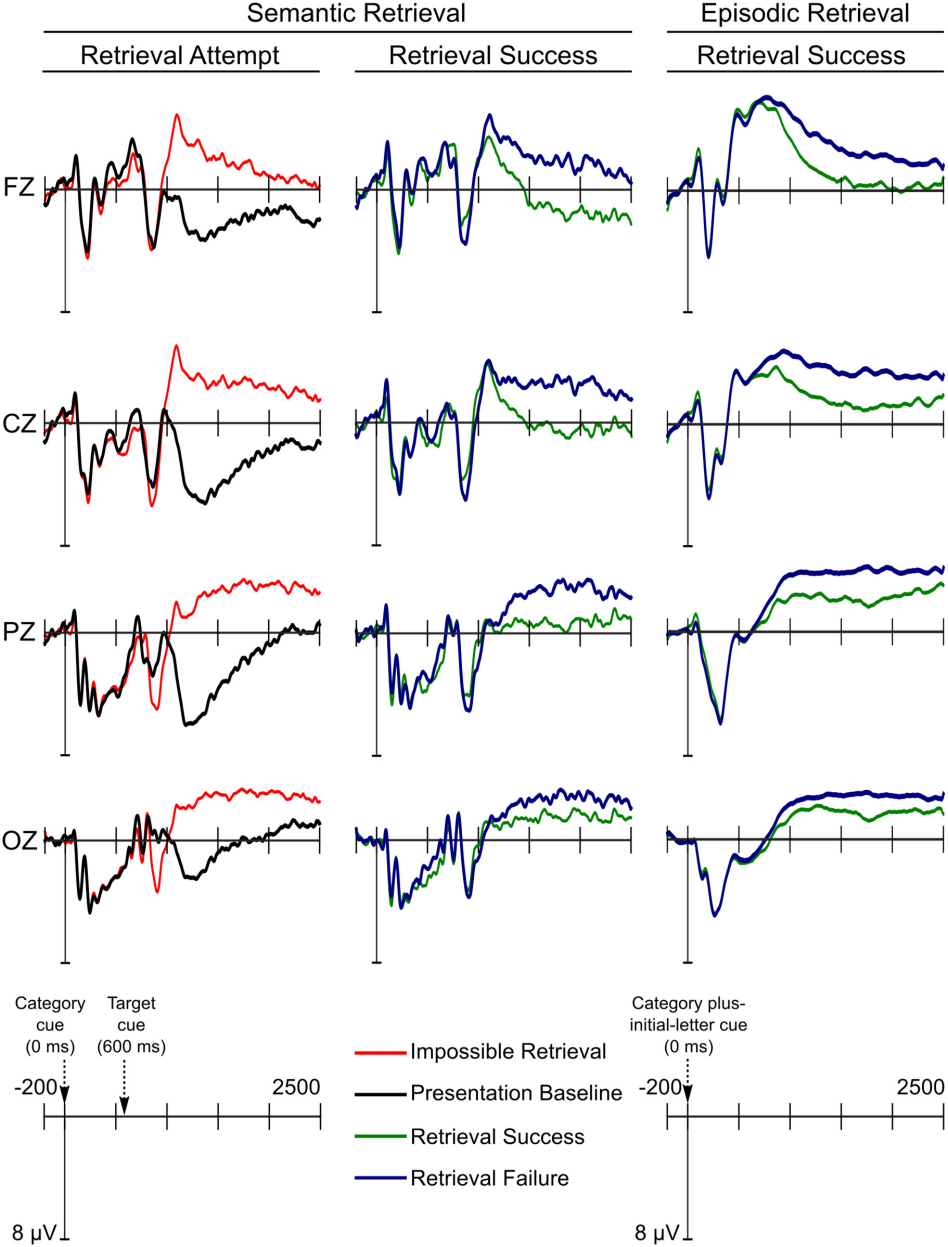
ERP amplitudes during the competitive semantic retrieval phase. Grand average ERPs from the four midline electrode sites are displayed for the retrieval attempt and the retrieval success comparisons.

**Fig 4:**
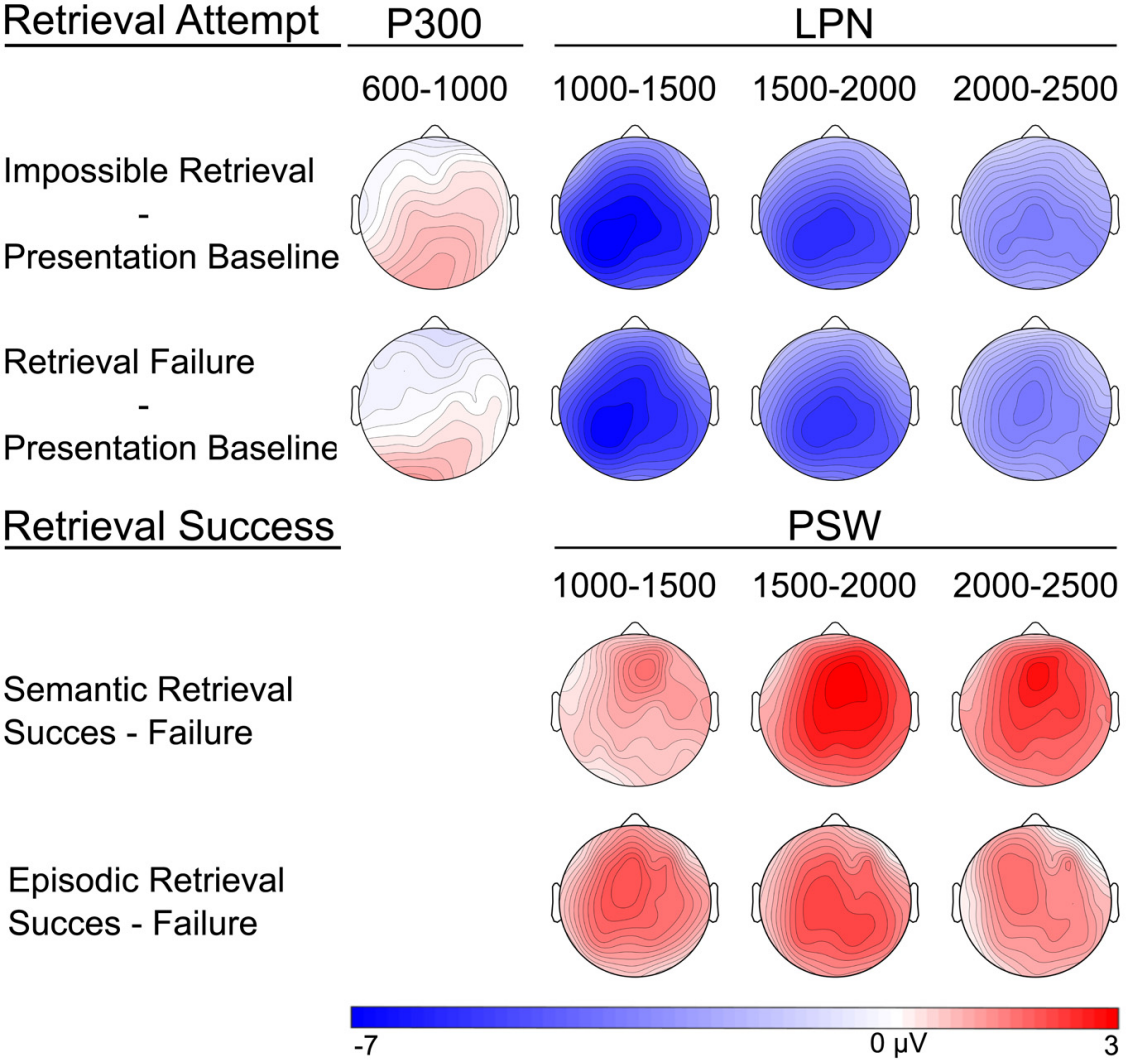
Topographical maps depicting the scalp distribution of amplitude differences in the word-stem cue time windows.

Retrieval attempt sensitive ERP effects should be evident in contrasts between unsuccessful retrieval attempts (both the impossible retrieval condition and retrieval failure trials in the possible retrieval condition) and the presentation baseline condition. As depicted in Figs 3 and 4, the earliest retrieval attempt effect was a positive going peak in the unsuccessful retrieval conditions compared with the presentation baseline condition over posterior electrode sites approximately 900 ms into the recording epoch (300 ms after word-stem cue presentation). This positive peak was followed by an anterior negative peak in the impossible retrieval and retrieval failure ERPs compared with the presentation baseline ERPs that was maximal approximately 1100 ms into the epoch (500 ms post word-stem cue presentation). As illustrated in Fig 3, this anterior peak was followed by a posterior negative slow wave in the impossible retrieval and retrieval failure conditions compared with the presentation baseline onsetting approximately 1100 ms into the recording epoch (500 ms after word-stem cue presentation) and lasting until the end of the recording epoch.

The statistical analyses indicated that the posterior positive peak was significant in the 600–1000 ms time-window (0–400 ms after the presentation of the word-stem cue) and that it was maximal over occipital regions (Fig 2). This P300-like effect did not correlate with RIF (neither in the impossible retrieval condition nor for the retrieval failure trials; all *p*s ≥ .198).

The anterior negative peak was maximal approximately 1100 ms after the presentation of the category cue (500 ms after the presentation of the word-stem cue; Fig 3). We conducted a peak-to-peak analysis to quantify the amplitude of the anterior negative peak effect. This effect might share functional significance with the N2 effect described in the Introduction, and thus reflect inhibitory control. The reason for conducting a peak-to-peak analysis was to isolate the negative peak from the temporally overlapping late posterior negative slow wave. First, we detected the local peak-amplitude [26] of the positive peak in the 800–1000 ms time window and of the negative peak in the 900–1200 ms time window for both the impossible retrieval condition and the presentation baseline condition for each participant. A sample was considered to be the local peak if it was the most extreme value (the most positive for the positive peak and the most negative for the negative peak) that was a) more extreme than the preceding and the following samples and b) more extreme than the average of the three preceding samples and the average of the three following samples. Next, we calculated a peak-to-peak score by subtracting the negative peak amplitude from the positive peak value separately for the impossible retrieval condition and the presentation baseline condition. To test if the peak-to-peak difference was larger in the impossible condition, we conducted a Condition (impossible retrieval/presentation baseline) one-way repeated measures ANOVA including data from electrode CZ (where the negative peak was maximal in amplitude). There was a main effect of Condition (*F*(1,17) = 20.872, *p* < .001, η^2^_p_ = .551), indicating that the peak-to-peak difference was indeed larger in the impossible retrieval condition compared with the presentation baseline condition (see Fig 3). Finally, we tested if the negative peak was related to forgetting, by correlating the peak-to-peak difference score and RIF in the impossible retrieval condition at electrode CZ. There was no correlation between the peak-to-peak measure and RIF (*r*_s_ = -.252, *p* = .313).

Finally, the statistical analyses showed that the late posterior negativity (LPN) effect was significant from the 1000–1500 ms time window until the end of the recording epoch. As illustrated in Figs 2 and 4, the LPN was maximal over left parietal electrode sites. Neither the impossible retrieval LPN nor the retrieval failure LPN correlated with RIF (all *p*s ≥ .053).

#### Semantic retrieval success

We reasoned that ERP correlates of semantic retrieval success should be evident in contrasts between retrieval success and retrieval failure ERPs within the possible retrieval condition. As predicted and as depicted in Fig 3, successful semantic retrieval was associated with an anterior positive slow wave (PSW), starting after approximately 1100 ms (500 ms post presentation of the word-stem cue) and persisting until the end of the recording epoch. The statistical analyses confirmed that this effect was significant from the 1000–1500 ms time window until the end of the recording epoch. As depicted in Figs 2 and 4, this PSW effect was maximal at frontal electrode sites. Contrary to predictions from the associative blocking account, there was no correlation between the ERP correlate of retrieval success, i.e. the PSW ERP effect, and RIF (all *p*s ≥ .088).

#### Episodic retrieval success

Next, we investigated the ERP correlates of episodic retrieval success by comparing retrieval success and retrieval failure ERPs from the final episodic test phase. Similarly, to semantic retrieval success, episodic retrieval success was reflected in a positive slow wave that started approximately 800 ms after cue presentation and persisted until the end of the recording epoch. The statistical analyses confirmed that this PSW effect was significant from the 1000–1500 ms time window until the end of the epoch. The effect was maximal over central electrodes in the 1000–1500 ms time window, parietal electrodes in the 1500–2000 ms time window and widespread in the final 2000–2500 ms time window (Figs 2, 3 and 4).

#### Comparison between the topographic distribution of the semantic and the episodic retrieval success effects

Finally, we investigated the possibility that separate neural sources underlies semantic and episodic retrieval by testing if the semantic and episodic retrieval success effects had distinct topographies in the three time windows where both effects were significant (1000–1500, 1500–2000 and 2000–2500 ms). Vector-scaled differences for the semantic and episodic retrieval success effects were first computed in each time window to control for differences in source strength [28]. Next, we performed a Retrieval type (semantic/episodic) x Electrode position (F3/FZ/F4/C3/CZ/C4/P3/PZ/P4/O1/OZ/O2) repeated measures ANOVA for each time window. There was no reliable interaction between Retrieval type and Electrode position in any of the three time windows (all *p*s ≥ .473).

## Discussion

The phenomenon that competitive retrieval can cause forgetting has been intensively studied for the last 20 years, but there is still an on-going debate of whether such forgetting is caused by associative blocking or inhibition. The present experiment contrasted these two theoretical accounts by investigating the extent to which RIF is contingent upon target retrieval by employing behavioural and electrophysiological methods. Besides investigating the neurocognitive mechanisms underlying RIF, we extend previous literature by isolating the ERP correlates of retrieval attempt and retrieval success in a semantic competitive-retrieval task.

### Is RIF Dependent of Target Retrieval?

Replicating Storm and colleagues [6], [7], the behavioural results indicate that RIF is independent of successful target retrieval by demonstrating RIF in an impossible retrieval task. This finding suggests that RIF is independent of target retrieval and is in line with the inhibitory control account of RIF. Although this finding is consistent with the inhibitory control account it is not necessarily inconsistent with the associative blocking account since it is possible that the participants covertly retrieve memories that do not match the cue in the impossible condition (e.g. [29]). These covertly retrieved non-targets could potentially block retrieval of the studied items in the final episodic test and hence cause RIF even in the absence of target retrieval.

There was no correlation between target retrieval in the competitive semantic retrieval phase and RIF. This finding does not support the predictions from the associative blocking account, which predicts that RIF is dependent on target retrieval. On the other hand, our measure of memory performance is limited to overt target retrieval and it is still possible that covert retrieval of task irrelevant memories (that do not match the retrieval cue) may correlate with RIF as would have been predicted by the associative blocking account.

Unexpectedly, we did not observe any correlations between RIF and the ERP correlates of retrieval attempt or retrieval success in the present study, making the the ERP results inconclusive regarding the role of target retrieval in RIF.

The question of whether RIF is dependent of strengthening of the association between the target and the retrieval cue has been examined with a different approach in a recent meta study [30]. In line with the behavioural results of the present experiment, this meta-analysis showed that increased memory performance for targets in the final test (their operationalization of strengthening) did not predict RIF in studies that had controlled for output interference in the final test. Output interference refers to the finding that the probability of retrieval success is dependent on output order in the memory test [31]. More specifically, items that are tested in the initial trials of the memory test are more likely to be remembered than items tested in later trials. If category cues are provided in the final test in the retrieval-practice paradigm, participants tend to retrieve the recently encountered practiced exemplars first, leading to output interference and lowered performance for non-practiced exemplars from the same category. Output interference can be controlled in the retrieval-practice paradigm if item-specific cues that dictate the output order are given in the final test, like the category-plus-initial-letter cues used in the present study.

To summarize, the behavioural results in the present study suggest that RIF is independent of target retrieval. This result supports the inhibitory control account, but does not necessarily rule out the associative blocking account, since covert retrieval of task irrelevant memories may cause RIF in the impossible condition. The ERP results are inconclusive regarding the role of target retrieval in RIF given the absence of correlations between the ERP effects and RIF. In addition to investigating the role of target retrieval in RIF, the present study allowed new insight into the ERP correlates of retrieval attempt and retrieval success in competitive semantic cued-recall.

### ERP Correlates of Semantic Retrieval Attempt

We included the impossible retrieval condition for two reasons. First, it enabled us to isolate retrieval attempt from retrieval success, since target retrieval was impossible and erroneous responses that did not match the cue were excluded in this condition (unlike the possible retrieval condition in which erroneous responses were coded as retrieval failure trials). Second, as a consequence of excluding incorrect responses, the impossible retrieval condition made it possible to investigate continuous retrieval attempts throughout the recording epoch, whereas the retrieval attempts were discontinued in the possible retrieval condition if the participant retrieved the target (retrieval success trials) or retrieved an erroneous exemplar (which potentially could occur in retrieval failure trials).

The earliest retrieval-attempt-related effect was a positive deflection in all of the retrieval conditions (including the impossible retrieval condition and retrieval failure trials) compared with the presentation baseline condition that peaked approximately 300 ms after the presentation of the word-stem cue (900 ms into the recording epoch). Such P300-like effects have previously been related to the allocation of attentional resources and may consequently indicate the allocation of attention to the relatively more difficult semantic cued-recall task compared with the reading task in the presentation baseline condition [32].

The P300 effect was followed by an N2-like effect. Similar N2 effects have previously been suggested to reflect inhibitory control during memory retrieval (for reviews see [33], [34]). We predicted that the N2 would correlate with RIF in the final test. Unexpectedly, however, there was no such correlation. The N2 may still reflect more general control mechanisms that are common to both retrieval conditions. The presentation of the word-stem cue is likely to lead to reactivation of memories that begin with the presented letters, but that do not belong to the category. In line with the pattern of results, such reactivation should occur both in the possible and impossible retrieval conditions, but not in the presentation baseline condition. The negative peak could hence be related to the detection of response conflict or to ensuing control triggered by such conflict. This interpretation holds that the negative peak should be related to forgetting of memories that begin with the same first two letters, but not with forgetting of semantically associated memories. The present experiment was however optimized for investigating retrieval competition based on semantic rather than orthographic association, so it is not possible to test this interpretation of the negative peak in the present experiment.

As expected, retrieval attempts gave rise to an LPN effect. This effect has previously been related to retrieval of attribute conjunctions, such as item-source and item-context associations, in episodic memory paradigms [22]. In the present study, the LPN may reflect retrieval of associated lexical representations during construction of candidates for cue-completion. Another prominent interpretation of the LPN is that it reflects action monitoring, including error-detection and conflict monitoring [22]. This interpretation is based on findings showing that the LPN is larger in recognition tasks involving response conflict. For example, false recognition of lures that are semantically related to studied items comes with longer response times and greater LPN amplitudes than true recognition of the studied items [35]. Interestingly, previous research has demonstrated that the false recognition LPN in stimulus-locked ERPs can be accounted for in terms of a phasic negative component revealed in response-locked analysis [35]. Although slightly more posterior, this response locked negativity resembles the error-related negativity (ERN) component thought to reflect action-monitoring mechanisms (e.g. [36], [37]), further suggesting that the LPN is related to error-detection or conflict monitoring in relation to the response. It is conceivable that the LPN in the current study reflects similar error-detection and retrieval monitoring when comparing a retrieved candidate with the retrieval cue. The two LPN interpretations should be considered complementary and are both likely to characterize the competitive semantic retrieval in the present study. Both the retrieval of attribute conjunction and the retrieval monitoring interpretations are consistent with the finding that the LPN was reduced after successful retrieval (see Fig 3). During unsuccessful retrieval trials (impossible condition and retrieval failure trials), the participants may have continued to construct new candidates that potentially complete the cue until the end of the trial. Similarly, during unsuccessful retrieval trials the participants may have continuously detected conflict between the constructed exemplars and the cue. When retrieval was successful there was no longer any need for retrieval of semantically associated lexical representations and construction of exemplars and consequently no longer any detection of conflict. The temporal overlap between the LPN and the more anterior retrieval success PSW effect complicates the interpretation of the attenuation of the LPN. The amplitude difference between conditions could be driven by one of these ERP modulations or both.

Both retrieval of attribute conjunctions and retrieval monitoring are expected to be iterative and their ERP correlates in averaged EEG should hence be tonic in line with the LPN effect in the present study. An important difference between the two interpretations of the LPN effect is that the retrieval of attribute conjunctions interpretation predicts that the LPN should *precede* semantic retrieval independently of whether the retrieved item was correct or not, whereas the error-detection interpretation predicts that the LPN should only be present *after* retrieval of exemplars that do not match the cue. We instructed the participants to withhold their response until the question mark appeared on the screen (after the ERP recording epoch had ended) to reduce the amount of muscle artefacts in the recording epoch. The participants did hence not respond within the ERP recording epoch in the present study, so we can not examine the relative contribution of the two processes to the LPN in the present study.

Both retrieval of attribute conjunctions and the error-detection interpretations can be regarded as essential parts of memory search processes. The finding that long-term memory search is related to posterior activity is in line with the attention to memory model [38]. According to this model, the parietal cortex is involved in directing attention towards relevant long-term memory representations. The LPN has previously been suggested to reflect these processes and has been related to parietal lobe activity in two separate ERP and fMRI studies using the same selective episodic retrieval paradigm [39], [40] and in a multimodal (fMRI, EEG and MEG) episodic memory study [41].

### ERP Correlates of Semantic and Episodic Retrieval Success

Both semantic and episodic retrieval success were reflected in a PSW effect. This effect started approximately 300 ms earlier for semantic (500 ms post word-stem cue presentation) compared with episodic retrieval (800 ms post category-plus-initial-letter cue presentation). The difference in onset-latency may be due to differences in cue-presentation procedures between tasks (sequential presentation of category and word-stem in the competitive semantic retrieval task compared with simultaneous presentation of category and initial-letter in in the final episodic test, see Fig 1) or be due to differences in difficulty (performance was lower in the episodic test). Differences between the two tasks are discussed in more detail in section 4.4 below.

The results are in line with previous studies of episodic cued recall that have related PSW effects with similar onset-time, duration, and topography to retrieval success (e.g. [12], [14], [16–19]). In general, the timing of the semantic retrieval success PSW in the present study is also in line with prior studies on retrieval success in semantic cued recall [5], [20]. It does however differ from the study by Cansino and colleagues where the PSW effect did not onset until 1000 ms post cue presentation. This difference in onset latency is likely due to task differences; rearranging letters until they combine into a word, as done in the scrabble task versus word-stem or word fragment completion tasks. Moreover, the PSW was right lateralized in the study by Cansino et al., but is typically bilateral, further suggesting that the scrabble task diverges from the tasks used in other studies.

The anterior topography of the semantic PSW effect in the present study is consistent with the observations in studies of episodic cued recall and with the semantic word-fragment completion task in Rass et al (2010). Surprisingly, the topography of the PSW was however more anterior in the present study compared with our previous study, using the same task [5]. Although the effect was significant all over the scalp in both experiments, it had an anterior maximum in the present study and a more posterior maximum in the previous study. Determining the topography of the PSW effect is complicated by the temporal overlap with the LPN. The present study diverges from the previous one in that half of the word stems were incompletable. Given that the current task was much more demanding and that the participants were unaware that impossible word-stems were included, they may have engaged in more retrieval monitoring in general in the present study. The difference in topography of the semantic retrieval success PSW effects in the two studies may hence be due an increment in the overlapping LPN component in the present study.

### Comparison between Semantic and Episodic Retrieval

The results in the present study suggests that overlapping neurocognitive processes are recruited during retrieval from the semantic and the episodic memory system. Successful retrieval was reflected in a PSW effect with similar timing and topography in both semantic and episodic retrieval. We primarily designed the present study to investigate the role of target retrieval in RIF and it should be noted that a side effect of optimizing the RIF effect was that the semantic and episodic retrieval tasks differed in procedures and difficulty. First, while the category and word-stem cues were presented sequentially in the competitive semantic retrieval phase they were presented simultaneously in the test phase (Fig 1). The reason for presenting the cues simultaneously in the final episodic test was to reduce interference in this phase of the experiment and thereby get a purer measure of RIF. Second, the participants were given a two letter word-stem cue in the competitive semantic retrieval phase compared with a one-letter-cue in the episodic final test phase, making the episodic test more difficult. Both these factors may limit the comparison between the ERP correlates of semantic and episodic cued recall in the present study.

Besides the PSW the present study also provides novel data suggesting that the LPN is involved in semantic retrieval. The LPN has previously been reported in episodic cued recall and recognition memory paradigms. The present study extends current literature by reporting an LPN in a semantic retrieval task, and suggests that the LPN reflects processes that are involved in both episodic and semantic retrieval.

To further investigate similarities and differences between episodic and semantic cued recall future studies should compare the ERP correlates of retrieval attempt and retrieval success in both memory systems within the same experiment using the same cueing procedure, similarly to how episodic and semantic retrieval tasks have been compared in recognition memory by contrasting recognition tasks and semantic judgement tasks (e.g. [42–44].

## Conclusions

The present study investigated the time course of competitive semantic retrieval and the neurocognitive mechanisms underlying RIF, in particular the role of target retrieval. The behavioural results replicate previous findings and suggest that RIF is independent of target retrieval. The ERP results are however inconclusive regarding the role of target retrieval in RIF. Besides informing theories of forgetting, the present study also isolated the ERP correlates of retrieval attempt and retrieval success in a competitive semantic retrieval task. Retrieval attempt gave rise to an LPN effect whereas retrieval success was evident in a PSW effect. Furthermore, we compared ERP correlates of retrieval success from semantic and episodic memory. The results suggest that similar cognitive processes are involved in retrieval from these two declarative long-term memory systems.

## Supporting Information

S1 Dataset Behavioural and ERP data. (XLSX)
